# Cannabinerol Restores mRNA Splicing Defects Induced by β-Amyloid in an In Vitro Model of Alzheimer’s Disease: A Transcriptomic Study

**DOI:** 10.3390/ijms26073113

**Published:** 2025-03-28

**Authors:** Maria Lui, Stefano Salamone, Federica Pollastro, Emanuela Mazzon, Osvaldo Artimagnella

**Affiliations:** 1 IRCCS Centro Neurolesi “Bonino-Pulejo”, Via Provinciale Palermo, Contrada Casazza, 98124 Messina, Italy; 2 Department of Pharmaceutical Sciences, University of Eastern Piedmont, Largo Donegani 2, 28100 Novara, Italy; salamone.ste@gmail.com (S.S.); federica.pollastro@uniupo.it (F.P.); 3 Department of Medical, Oral and Biotechnological Sciences, University “G. D’Annunzio” Chieti-Pescara, 66100 Chieti, Italy

**Keywords:** cannabinerol, phytocannabinoids, alternative splicing analysis, transcriptomics, Alzheimer’s disease, intron retention, miRNA targeting

## Abstract

Alzheimer’s disease (AD) is the most common form of dementia, characterized by β-amyloid (Aβ) plaques and neurofibrillary tangles, leading to neuronal loss and cognitive impairments. Recent studies have reported the dysregulation of RNA splicing in AD pathogenesis. Our previous transcriptomic study demonstrated the neuroprotective effect of the phytocannabinoid cannabinerol (CBNR) against the cell viability loss induced by Aβ in differentiated SH-SY5Y cells. This study also highlighted the deregulation of genes involved in mRNA splicing after Aβ exposure or CBNR pre-treatment. Here, we investigated whether CBNR could restore the splicing defects induced by Aβ in an AD in vitro model. Using the rMATS computational tool for detecting differential alternative splicing events (DASEs) from RNA-Seq data, we obtained 96 DASEs regulated in both conditions and, remarkably, they were all restored by CBNR pre-treatment. The pathway analysis indicated an over-representation of the “Alzheimer’s disease–amyloid secretase pathway”. Additionally, we observed that Aβ exposure increased the frequency of retained introns (RIs) among the shared DASEs, and that this frequency returned to normality by CBNR pre-treatment. Interestingly, most of these RIs contain a premature in-frame stop codon within the RNA sequence. Finally, analyzing the DASE regions for miRNA hybridization, we found 33 potential DASE/miRNA interactions that were relevant in AD pathogenesis. These findings revealed a novel trans-gene regulation by CBNR, potentially explaining part of its neuroprotective role. This is the first study demonstrating the involvement of a cannabinoid in the regulation of mRNA splicing in an AD model.

## 1. Introduction

Understanding the molecular mechanisms underlying the pathological dysfunction of Alzheimer’s disease (AD) is a fundamental step, given the lack of a cure. AD is a neurodegenerative disorder neuropathologically characterized by extracellular β-amyloid (Aβ) plaques and intracellular neurofibrillary TAU-containing tangles and, clinically speaking, by a progressive decline in memory. Recently, a genome-wide association study (GWAS) of sporadic AD patients identified around 75 different genes/loci, suggesting the influence of multiple biological processes on the pathogenesis of AD [1]. In addition to the GWAS, transcriptomic and proteomic profiles of postmortem brain tissue were obtained from a recent large cohort study (ROSMAP), which highlighted alterations in the gene expression of AD-related risk factor genes [2]. The biological domains affected by AD include Aβ generation and clearance, TAU proteostasis, neuronal apoptosis, mitochondrial and metabolic deficits, synaptic loss, and oxidative stress (as reviewed in [3]). Interestingly, among the biological pathways altered in AD, alternative mRNA splicing has emerged as a key regulatory mechanism.

Alternative splicing (AS) is a genome-wide post-transcriptional mechanism that allows one pre-mRNA molecule to produce multiple distinct mRNA isoforms, contributing to the functional diversity and complexity of proteins expressed in tissues, especially in the nervous system [4]. AS arises from the combination of cis-regulatory elements and trans-acting factors, and different signaling pathways have been observed to influence AS [5,6]. The mis-regulation of AS has been associated with numerous diseases [7,8], including AD [9]. Initial reports were focused on the main AD-associated genes, such as *APOE*, *APP*, *BACE1*, *MAPT*, and *PSEN1*-2 (as reviewed in [10]). However, the accumulation of the ribonucleoprotein components of the spliceosome into insoluble fractions, as well as the close association with neurofibrillary tangles in post-mortem AD brains [11,12,13], suggest that aberrant splicing events may be pervasive, leading to the onset and progression of AD pathology.

It has been reported that *TAU* gene expression alteration generated splicing errors in human neuronal culture and fly models, including intron retention and non-annotated cryptic splice junctions [14]. Interestingly, retained intron events have been shown to increase during aging. The differential analysis of retained intron (RI) events in aging Drosophila and human brain tissues showed that genes with intron retention are involved in AD-related pathways [15]. Moreover, in the AD frontal cortex, more than one hundred differential RI genes showed altered levels of protein expression compared to the control samples [15]. The retention of an intron may include a premature stop codon in the open reading frame (ORF) [16], or it may become a new interface potentially targeted by microRNAs (miRNAs), long non-coding RNAs (lncRNAs), and/or RNA-binding proteins, ultimately affecting the post-transcriptional regulation of the gene [17,18]. Finally, in humans, deep RNA-sequencing from ROSMAP individuals revealed hundreds of aberrant pre-mRNA splicing events associated with AD [19]. Altogether, these findings provide evidence that the dysregulation of mRNA splicing is an important feature of AD.

In the light of this evidence, the discovery of novel compounds that may contrast aberrant splicing regulation in AD could lead to a new phase in translational and clinical medicine to overcome the actual lack of efficacious therapies available to treat AD patients. In this regard, derivatives of *Cannabis sativa*, namely cannabinoids, have recently been demonstrated to be neuroprotective agents against AD. For example, Δ^9^-tetrahydrocannabinol (Δ^9^-THC), cannabidiol (CBD), and cannabigerol (CBG; all three are abundant and well-studied phytocannabinoids) reduced Aβ accumulation and toxicity in in vitro and in vivo AD models (as reviewed in [20,21,22]). Our group previously documented how some “minor” phytocannabinoids, such as ∆^8^-tetrahydrocannabinol (Δ^8^-THC) and cannabinerol (CBNR), also improve the cell viability of neuronal cultures exposed to Aβ. We demonstrated that Δ^8^-THC modulates the genes involved in endoplasmic reticulum stress and proteostasis [23]; and that CBNR upregulates the genes related to mitochondrial oxidative phosphorylation, protein folding and degradation, and glucose and lipid metabolism [24]. However, whether cannabinoids may act on the AS process has not yet been investigated in detail. A recent multi-omics study reported that, in the prefrontal cortex, a synthetic cannabinoid (WIN 55,212-2) regulated many alternative splicing events [25].

In this work, we investigated whether CBNR pre-treatment may counteract aberrant AD splicing defects in an in vitro AD model. An mRNA splicing pipeline, starting with transcriptomics sequencing data, was used to detect DASEs, further integrating the prediction analysis of DASE/miRNA hybridization and DASE/lncRNA mapping. This is the first study investigating the impact at the omics level of a cannabinoid on mRNA splicing regulation mechanisms, with the aim of preventing neuronal AD dysfunctions.

## 2. Results

### 2.1. CBNR Pre-Treatment Regulates Splicing-Related Biological Processes

In a previous study [24], we sequenced the transcriptome of retinoic acid (RA)-differentiated SH-SY5Y neurons that were treated with 10 µM Aβ and pretreated with 20 µM CBNR (CBNR20+Aβ sample), treated with Aβ alone (Aβ sample), or acting as a control (CTR sample). Here, we retrieved the differentially expressed genes (DEGs) obtained by comparing the CBNR20+Aβ sample against the Aβ sample (CBNR20+Aβ vs. Aβ), and the Aβ sample against the CTR sample (Aβ vs. CTR). Starting from these DEGs, we performed a GO over-representation analysis of the biological processes (BPs) in both comparisons, with the aim of highlighting which BPs may be counteracted by CBNR pre-treatment in an in vitro AD model.

**Figure 1:**
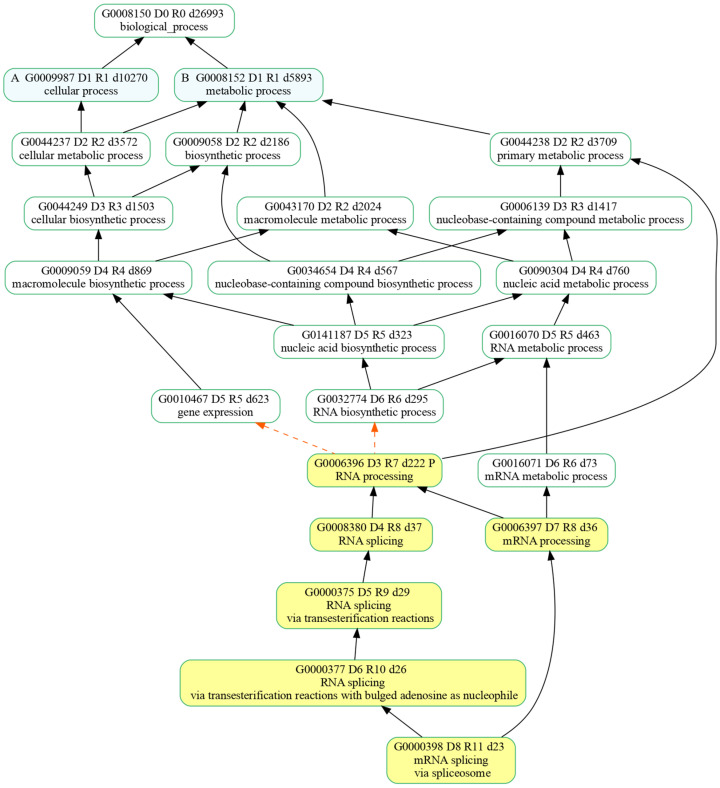
Directed acyclic graph representing enriched splicing-related biological processes and their relationships. The nodes highlighted in yellow identify the biological processes that were enriched for both the comparisons (Aβ vs. CTR and CBNR20+Aβ vs. Aβ) with the overall high-fold enrichment score. Formal relationships among processes are represented by either solid black arrows or dashed orange arrows, respectively, for the “is a” and “part of” attributes.

The PANTHER GO over-representation analysis (ORA) showed 299 and 86 over-represented significant BPs in the Aβ vs. CTR and CBNR20+Aβ vs. Aβ comparisons, respectively (Table S1). Of these, we focused on the 63 BPs that were significantly over-represented in both comparisons. Interestingly, among these GO terms, the ones related to mRNA splicing had the highest fold enrichment, as reported in Table 1. A GOATOOLS analysis of the splicing-related BPs revealed the formal relationships among them through a directed acyclic graph (Figure 1). The mRNA splicing process appeared to be influenced by both Aβ exposure and CBNR pre-treatment, suggesting that they regulate alternative splicing patterns.

### 2.2. Effects of CBNR Pre-Treatment on Alternative mRNA Splicing

In order to investigate the mRNA splicing regulation exerted by CBNR pre-treatment in AD, we conducted a transcriptomic splicing analysis, quantifying the differential alternative splicing events (DASEs) in Aβ vs. CTR and CBNR+Aβ vs. Aβ. Their sequenced transcriptomes were used to detect and quantify DASEs using the rMATS (replicate Multivariate Analysis of Transcript Splicing) computational tool [26] in five different types of events, including skipped exon (SE), alternative 5′ (A5SS) and 3′ (A3SS) splice sites, retained intron (RI), and mutually exclusive exon (MXE). The tool computed how often a specific exon was included in a particular region and expressed this measure as PSI (Percent Spliced In). All DASEs were therefore detected and quantified with rMATS for both Aβ vs. CTR and CBNR+Aβ vs. Aβ (Table S2).

**Figure 2:**
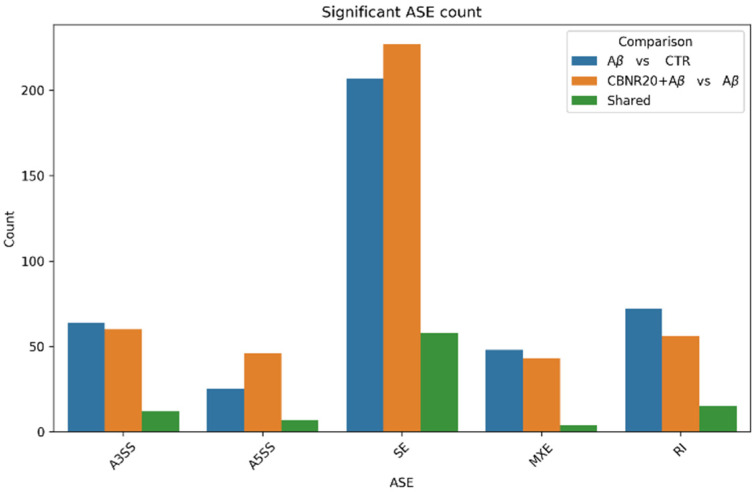
DASE count for each category resulting from rMATS analysis. We detected and quantified A5SS, A3SS, SE, and RI DASEs for Aβ vs. CTR (blue bars) and CBNR20+Aβ vs. Aβ (orange bars). The number of shared DASEs between the two comparisons is also illustrated (green bars).

We retained only DASEs whose PSI values were significantly different across the conditions (ΔPSI) and characterized by high mean coverage and significance level based on FDR. Specifically, in Aβ vs. CTR, rMATS detected 76 A3SS, 32 A5SS, 52 MXE, 87 RI, and 265 SE events, while in CBNR+Aβ vs. Aβ, it revealed 72 A3SS, 53 A5SS, 47 MXE, 71 RI, and 285 SE events. Interestingly, we found 96 DASEs shared between the two comparisons that were found to be significantly different in the Aβ samples with respect to the CTR samples, as well as the CBNR20+Aβ samples compared to the Aβ samples (shared DASEs). They specifically consisted of 12 A3SS, 7 A5SS, 4 MXE, 15 RI, 58 SE, which were further investigated, as they referred to 87 genes that were possibly involved in common pathways. The number of DASEs for each type of splicing event and for each comparison is summarized in Figure 2, further highlighting the relative abundance of shared DASEs across the two comparisons. The de novo counts of each DASE category for both comparisons are reported in Figure S1.

### 2.3. CBNR Pre-Treatment Counteracts Aβ-Induced mRNA Splicing Defects

To determine whether CBNR pre-treatment was able to restore the splicing events caused by Aβ treatment to a condition more similar to the control, we specifically focused on the 96 shared DASEs characterizing 87 different genes. We therefore aimed to evaluate which biological processes and pathways were influenced by this set of genes. For this purpose, we performed Gene Ontology (GO) and PANTHER Pathway over-representation analyses. The PANTHER Pathway analysis results are reported in Table 2 and Table S3; interestingly, they show only the “Alzheimer’s disease–amyloid secretase pathway” as statistically significant (FDR < 0.05), whereas no significant biological processes were found in the GO over-representation analysis (Table S3).

**Figure 3:**
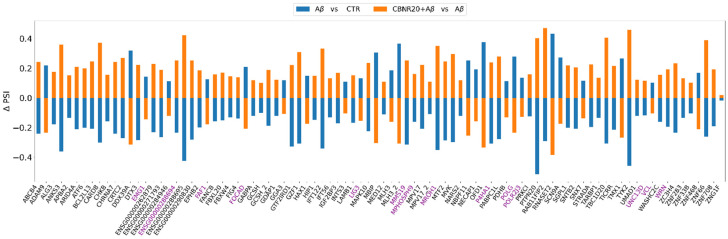
Histogram plot of each shared DASE ΔPSI value for Aβ vs. CTR (in blue) and CBNR20+Aβ vs. Aβ (in orange). In violet are the genes in which a de novo (unannotated) splicing variant has been detected.

**Figure 4:**
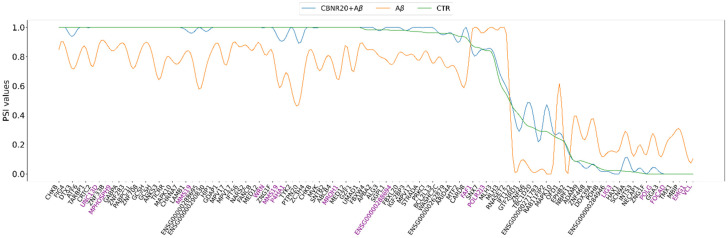
Line plot of the shared DASE PSI values for each investigated sample condition. CTR (in green), Aβ (in orange), and CBNR20+Aβ (in blue). In violet are the genes in which a de novo (unannotated) splicing variant has been detected.

Moreover, the shared DASEs were further investigated for their ΔPSI and absolute PSI values to identify common patterns or differences across the conditions. Curiously, all the shared DASEs demonstrated opposite ΔPSI in both comparisons, meaning that CBNR pre-treatment restored the splicing pattern dysregulation induced by Aβ (Figure 3). Moreover, in Figure 4, we highlight how the absolute PSI values of the CTR and CBNR20+Aβ samples are more similar to each other than the Aβ ones, confirming that CBNR pre-treatment restores the condition to the CTR level.

The results from rMATS analysis, both in terms of ΔPSI and absolute PSI values, allow us to hypothesize the potential of the phytocannabinoid to counteract the effect of Aβ and eventually restore conditions to those of the control group.

**Figure 5:**
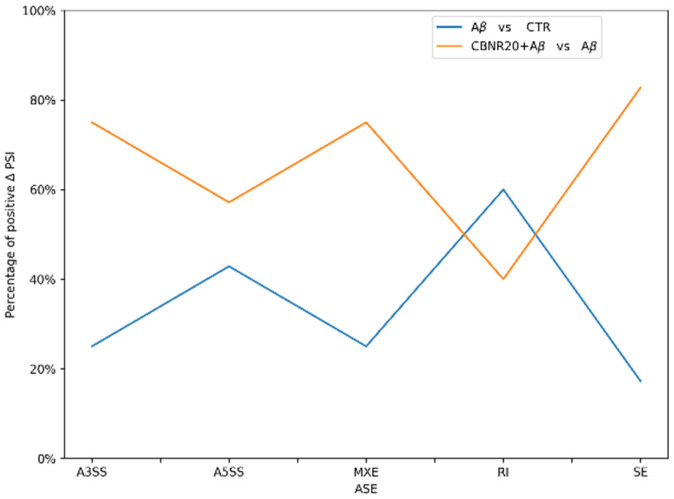
Line plot showing the percentage of ASEs with positive ΔPSI values for each comparison. The comparison of CBNR20+Aβ vs. CTR exhibits a greater number of events with positive ΔPSI values (orange line), indicating an enrichment of splicing changes in this condition. In contrast, the Aβ vs. CTR comparison is predominantly characterized by ASEs with negative ΔPSI values (blue line). Notably, this trend is reversed for RI events.

Notably, an evaluation of the percentage of events with a positive ΔPSI for each ASE type highlighted an opposite pattern for the two conditions, as shown in Figure 5. A majority of the events in the comparison with CBNR20+Aβ were characterized by positive ΔPSI, while Aβ vs. CTR were mainly affected by ASEs with negative ΔPSI. This trend was followed by the A3SS, A5SS, MXE, SE events but not the RI ones, which making the investigation of this type of event particularly interesting.

### 2.4. RI Events of Shared DASEs Include Premature Stop Codons

**Figure 6:**
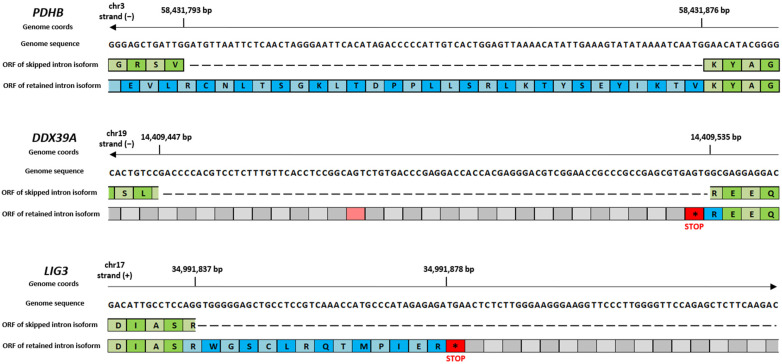
Examples of premature stop codon identification on RI events, obtained through manual IGV inspection. Three distinct examples of RI events in the *PDHB*, *LIG3*, and *DDX39* genes are shown. These events resulted in the following outcomes: no premature stop codons in *PDHB* (ORF frame 1), one premature stop codon in *LIG3* (ORF frame 2), and two premature stop codons in *DDX39* (ORF frame 3). Asterisk symbols in red boxes were used to indicate translation stop codons. Green boxes represent amino acids coded by canonical isoform, whereas the blue ones represent amino acid of the alternative isoform.

Based on the aforementioned results, we specifically investigated the RI DASE class to infer their ultimate effect on gene expression. RI events can either introduce new functional elements within mRNAs or lead to the introduction of premature termination codons, resulting in mRNA degradation by a surveillance mechanism called nonsense-mediated decay (NMD). Indeed, RIs are commonly associated with lower protein levels, caused by the inclusion of an in-frame stop codon and ultimately leading to a dramatic change in the expression of the gene affected by the splicing variant [27]. We therefore derived the nucleotide sequence of the retained introns and checked whether a stop codon occurred before the original termination codon. An examination of the RI fasta sequences revealed the presence of multiple premature stop codons for the majority of them, with the exception of *MROH1* and *PDHB*, which did not comprise any premature in-frame stop codons (Table S4). In Table 3, we summarize all the premature stop codons that were identified in each RI under investigation, considering the correct ORF manually identified using the IGV software (V. 2.18). Figure 6 shows three different examples of retained introns for the *PDHB*, *LIG3*, and *DDX39* genes.

### 2.5. DASE Regions Results to Be Targeted by Brain- and AD-Associated miRNAs

**Figure 7:**
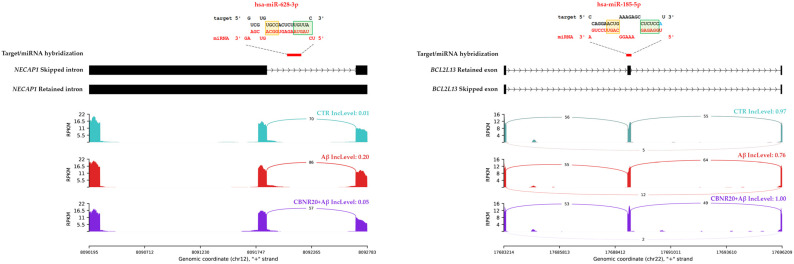
Sashimi plots illustrating two miRNA hybridizations on *NECAP1* (**left**) and *BCL2L13* (**right**) DASE regions. Both sashimi plots depict the read coverage for each sample and the corresponding PSI value. The DASE/miRNA alignment is illustrated accordingly, highlighting the seed (in green) and post-seed (in orange) regions. Adenine in the first position is colored in green (on the left).

Since DASE events can either introduce or remove new targeting sites for some non-coding RNAs (ncRNAs), we analyzed the shared DASE sequences for possible miRNA hybridization and lncRNA mapping, potentially regulating gene expression and function. For this purpose, we first performed a hybridization prediction analysis of 630 human miRNAs (deposited at MirGeneDB 2.1) with the 96 shared DASEs, using the RNAhybrid tool (V. 2.1.2). The analysis resulted in 21,121 different hybridizations (Table S5). To retain only the strong interactions, the DASE/miRNA hybridizations were filtered to obtain the 839 with an MFE (Minimum Free Energy) ≤ −30 kcal/mol and made of 61 DASE sequences that were predicted to interact with 207 miRNAs. The DASE/miRNA predicted alignments were ultimately classified based on seed and post-seed pairings and resulted in 70 “Canonical”, 11 “Strong”, and 56 “Compensatory” miRNA alignments (see in Section 4 for details). The remaining 702 hybridizations not falling under any of the three classes were discarded. Lastly, we decided to further investigate only the miRNAs whose elevated expression at the brain level was detected by the previous NGS experiments, taking advantage of the miRNATissueAtlas2 database. This approach narrowed our selection to 18 distinct miRNAs, which were found to be highly expressed in brain tissue and predicted to interact with 17 DASEs through 33 different hybridizations while exhibiting a very low MFE (two graphical representations of DASE/miRNA hybridizations are shown in Figure 7). As showed in Table 4, we finally obtained thirteen DASEs with negative ΔPSI in the Aβ vs. CTR comparison, while four DASEs had positive ΔPSI. As mentioned above, the opposite ΔPSI was observed in the CBNR20+Aβ vs. Aβ comparison. Three out of four ΔPSI > 0 DASEs in Aβ vs. CTR were specifically RI events (on the *ENSG00000284946*, *NECAP1*, and *POLG* genes) and one was an A5SS (on *POLR2J3* gene). Instead, among the ΔPSI < 0, we specifically obtained nine SE events (on *APBA2*, *BCL2L13*, *CHRNA7*, *FBXL20*, *FBXW4*, *HIP1*, *LAMB1*, *MVK*, and *ZC3H4*), two A3SS (on *GTF2IRD1*, and *TYK2*), and two RI (on *MAPK10*, and *MROH1*). Interestingly, most of the miRNAs predicted to interact with DASE sequences were highly associated with AD, according to the public database (10.1093/database/baae066), as reported in Table S5.

Lastly, we explored the possibility that the lncRNA antisense genes may be mapped within the shared DASE regions, contributing to their post-transcriptional gene regulation. For this purpose, we mapped 323,950 different lncRNAs transcripts (belonging to 95,243 different lncRNA genes; retrieved from the LncBook 2.0 database [28]) to the 96 shared DASE sequences, using the IntersectBed tool (V. 2.30.0) [29]. We obtained 68 different lncRNA antisense transcripts (belonging to 20 lncRNAs genes), potentially mapping to 18 different DASE regions (Table S6). To retain the brain-expressed lncRNAs, we adopted the NONCODE database, identifying only five lncRNAs expressed in the brain. Notably, *CHRNA7* and *MAPK10* genes resulted to be mapped by *HSALNG0035653* and *HSALNG0104832* lncRNA genes, respectively (Table 5). However, to date, this class of human ncRNAs is still not fully annotated for their tissue expression, making this type of analysis especially challenging.

Altogether, these data suggest that a fraction of the shared DASE sequences are probably targeted by the miRNAs and lncRNAs in neurons. Therefore, Aβ exposure may increase and decrease mRNA isoforms, leading to, respectively, the aberrant downregulation or upregulation of 17 genes involved in or putatively implicated in AD pathogenesis, whereas CBNR pre-treatment restored the normal splicing pattern of these genes, avoiding potential gene mis-regulation and dysfunction. Interestingly, from both the miRNA and lncRNA analyses, we noted that the *CHRNA7* and *MAPK10* genes, as well as the *APBA2* gene from the miRNA analysis alone, were all involved in the “Alzheimer’s disease–amyloid secretase pathway” (Table 2 and Table 5), corroborating the importance of their gene expression regulation in AD.

## 3. Discussion

The alternative splicing of pre-mRNA is a basic mechanism suitable for the generation of functional diversity from the same RNA molecules. mRNA isoforms may contain different coding and non-coding domains, which have different functional properties and targeting sites. GWAS, proteomic studies, and transcriptomic advances, have suggested that aberrant the AS of pre-mRNA contributes to AD pathogenesis [1,2,19]. The splicing mis-regulation of AD-associated genes, such as *APOE*, *APP*, *BACE1*, *MAPT*, and *PSEN1*-2, was initially reported (as reviewed in [10]). For instance, the AS of *MAPT* exon 10 results in isoforms with three (3R-tau) or four (4R-tau) microtubule-binding repeats; the disruption of the 3R-tau/4R-tau ratio due to mutations on exon 10 is sufficient to drive tauopathies [30]. Moreover, the retention of *MAPT* intron 11 generates a premature stop codon, leading to a truncated form of the TAU protein with altered biochemical properties, as evidenced in the prefrontal cortex of AD females [16]. Recently, a deep transcriptomic profiling of AD post-mortem brains revealed hundreds of deregulated splicing events, confirming how the AS process is highly associated with AD pathogenesis [19].

As known, to date, there is no definitive cure for AD; therefore, further understanding the molecular mechanisms underlying this pathology, and finding novel compounds that may also act on splicing regulation, is fundamental. In recent years, natural compounds have been tested for their intrinsic neuroprotective properties. Among them, cannabinoids have been shown to play antiapoptotic and antioxidant roles in in vitro and in vivo AD models [20,22]. However, no data are available about a possible role of phytocannabinoids in the regulation of AS and, in particular, in restoring the aberrant AS occurring in AD pathogenesis. Our group recently published an article on a novel “minor” phytocannabinoid, CBNR, showing how it may have interesting neuroprotective properties in an in vitro AD model [24].

In this study, we focused on the possibility that CBNR may counteract aberrant AD splicing defects, investigating its impact on mRNA splicing regulation at omics level. Based on the transcriptomic data that we recently obtained from RA-differentiated SH-SY5Y cells exposed to Aβ and pre-treated with CBNR 20 µM [24], we observed that Aβ (Aβ vs. CTR comparison) and CBNR pre-treatment (CBNR20+Aβ vs. Aβ comparison) were involved in mRNA splicing (Table 1, Figure 1), confirming that AS was affected in our AD model and suggesting that CBNR regulated the AS process and potentially contributed to final neuronal protection. In the light of these findings, we further investigated whether and which genes were regulated at the AS level, following the pre-treatment with CBNR in the AD model.

For this purpose, we took advantage of a well-standardized pipeline to detect and quantify DASEs using the rMATS tool [26]. Specifically, we obtained 87 genes regulated at the splicing level in both Aβ vs. CTR and CBNR20+Aβ vs. Aβ, counting a total of 96 shared DASEs (Figure 2), of which 14 have not been annotated yet (Figure S1). In this study, we focused our analysis on the shared events, with the aim of specifically understanding in which processes and how CBNR pre-treatment may counteract splicing defects in an AD model. Indeed, the pathway analysis revealed that the “Alzheimer’s disease–amyloid secretase pathway” was significantly over-represented (Table 2), suggesting that CBNR pre-treatment was able to restore the splicing pattern of key genes associated with AD, acting as a neuroprotective compound. The following five genes were included in the pathway: the ADAM Metallopeptidase Domain 9 (*ADAM9*) gene, which is involved in the α-secretase activity of the amyloid precursor protein (*APP*); the Amyloid Beta Precursor Protein Binding Family A Member 2 (*APBA2*) gene, a neuronal adapter that interacts with and stabilizes the APP; the Cholinergic Receptor Nicotinic Alpha 7 Subunit (*CHRNA7*) gene, which encodes for a subunit of the neuronal nicotinic receptor that mediates fast signal transmission at cholinergic synapses and which is highly expressed in the hippocampus, one of the main brain area affected in AD; the Mitogen-Activated Protein Kinase 10 (*MAPK10*) gene, which encodes for a serine-threonine kinase involved in a wide variety of cellular functions, including apoptosis, and which also phosphorylates APP, regulating its signaling; and the Protein Kinase C Iota (*PRKCI*) gene, another serine-threonine kinase that inhibits Aβ-induced apoptosis and negatively regulates autophagy. Overall, qualitatively speaking, the 87 genes obtained from the splicing analysis are mainly involved in protein localization and transport, DNA damage response, and DNA replication (Table S3).

Interestingly, the percentage of isoforms spliced in (ΔPSI positive) or spliced out (ΔPSI negative) the shared DASE events was always opposite in the two comparisons (Figure 3), suggesting that CBNR pre-treatment may restore the aberrancy induced by Aβ. Indeed, the absolute PSI values of the CTR and CBNR20+Aβ samples were similar compared to those of the Aβ samples (Figure 4), confirming that CBNR pre-treatment restored the condition to the CTR level. Moreover, in the average per ASE category, there were more spliced out events in Aβ vs. CTR (mainly in SE events) as compared to CBNR20+Aβ vs. Aβ. However, the RI events displayed an opposite behavior, with more introns retained in Aβ vs. CTR compared to CBNR20+Aβ vs. Aβ (Figure 5). These findings suggest that intron retention is an event frequently occurring in SH-SY5Y neurons exposed to Aβ, and that pre-treatment with CBNR restores the CTR condition. Finally, analyzing the RI nucleotide sequence for the stop codon screening, we found that all of them (except for the *MROH1* and *PDHB* genes) contain at least one in-frame stop codon (Table 3, Figure 6), meaning that the proteins derived from mRNAs containing these RIs will also incorporate a premature stop codon. Therefore, an RI could lead to a potential change in the function of the protein or missing and/or acquiring functional domains; the gene could also undergo the nonsense-mediated mRNA decay mechanism [31], ultimately downregulating gene expression level. This result is aligned with those of other papers which have reported the frequency and importance of RIs in the transcriptomic analyses of AD models and patients [15,19].

Altogether, these findings allow us to hypothesize the potential of this phytocannabinoidto counteract the effect of Aβ and, ultimately, return conditions to those of the control group.

Every RNA sequence involved in the AS process could be targeted and regulated by miRNAs, lncRNAs, and/or RNA-binding proteins. Moreover, it is well known that miRNA expression alterations are associated with AD pathogenesis (as reviewed in [32]). For this reason, we performed a target/miRNA predictive analysis and, in addition, we explored the potential impact of lncRNAs, an emerging aspect in AD [33].

Regarding the miRNA analysis, it involved all the 96 shared DASEs and aimed to find putative miRNAs that may bind to alternative RNA sequences, potentially regulating gene expression and function. AS can indeed create or remove miRNA-binding sites, depending on the splicing variants, or cause changes in miRNA binding by affecting the mRNA structure. This can eventually lead to enhanced or reduced miRNA binding thus inducing an over- or under-expression of the gene transcript. miRNAs are small non-coding RNAs (sncRNAs) of 22–23 nucleotides that target mRNA sequences and induce the repression of gene expression at post-transcriptional level. As is known, the canonical mechanisms include mRNA degradation and translational repression via the recruitment of Argonaute proteins. Target/miRNA interactions are highly heterogeneous, along with the mRNA regions targeted. Many reports have shown 3′UTRs as the main regions involved in miRNA interactions; however, to date, both 5′UTRs and CDSs are implicated in miRNA hybridization. Usually, the standard rules are as follows: Matching should occur at the level of the seed region (2–7 nt of miRNA), preferentially with an adenine at the first position, and matching at the post-seed region (13–16 nt). Pairings of both regions are often associated with a strong effect on mRNA downregulation, whereas the presence of loops within the seed region due to mismatches, compensated by perfect match at the post-seed region, may often be implicated in translation repression [34,35,36,37].

The DASE/miRNA hybridization analysis revealed that 18 miRNAs highly expressed in brain might target the DASE sequences belonging to 17 genes (Table 4, Figure 7). Thirteen DASEs had a negative ΔPSI in the Aβ vs. CTR comparison, while four DASEs had a positive ΔPSI. Interestingly, most of the miRNAs targeting these DASE sequences have been reported to be highly associated with AD [38].

Three out of four ΔPSI > 0 DASEs were RI events (on *NECAP1*, *POLG*, *ENSG00000284946* genes), and one was an A5SS (on *POLR2J3* gene). These data suggest that Aβ exposure increases mRNA isoforms with retained introns, harboring also a premature in-frame stop codon, and a long exon form at the 5′ site, which are targeted by putative miRNAs, thus leading to the downregulation of gene expression. Following CBNR pre-treatment, however, the isoforms including these regions decreased, avoiding the mis-regulation of genes. Concerning their functional role, *NECAP1* encodes for a protein localized to clathrin-coated vesicles involved in the endocytosis process. Mutations in *NECAP1* have been correlated with syndromic epilepsies [39]. Interestingly, the downregulation of *NECAP1* was found in AD post-mortem brains [40]. The *POLG* gene encodes for the catalytic subunit of mitochondrial DNA polymerase, suggesting that its downregulation impairs mitochondrial metabolism and function in AD [41,42,43]. Notably, a total of four miRNAs seem to target its RI DASE sequence. However, *ENSG00000284946* is a novel gene with unknown functions so far. Last, the *POLR2J3* gene, which contains an A5SS event, encodes for a subunit of the RNA polymerase II that synthesizes mRNAs [44]. miRNA targeting its sequence potentially leads to its downregulation and in turn affects global transcription.

Regarding the 13 DASEs with a negative ΔPSI in Aβ vs. CTR, we specifically obtained 9 SE (on *APBA2*, *BCL2L13*, *CHRNA7*, *FBXL20*, *FBXW4*, *HIP1*, *LAMB1*, *MVK*, and *ZC3H4*), 2 A3SS (on *GTF2IRD1*, and *TYK2*), and 2 RI (on *MAPK10*, and *MROH1*). The miRNA targeting of these DASEs suggests that, under AD conditions, they cannot be downregulated, whereas CBNR pre-treatment restores a normal splicing pattern of these genes, avoiding their potential upregulation and dysfunction. Remarkably, some of them are targeted by more than one miRNA, such as *APBA2*, which are involved in the “Alzheimer disease–amyloid secretase pathway” together with the *CHRNA7* and *MAPK10* genes (see above in Table 2), corroborating the importance of the regulation of their gene expression in AD. Other genes with more miRNAs include *TYK2*, *HIP1*, *MROH1*, *MVK*, and *ZC3H4*. Importantly, the upregulation of *TYK2*, *FBXL20*, and *BCL2L13* has been demonstrated to be correlated with AD pathological conditions. Specifically, *TYK2* gene encodes for a member of the Janus kinase (JAK) protein family, regulating cell growth and development. Recently, it has been also reported that TYK2 phosphorylates the TAU protein promoting its aggregation in human cells, while its downregulation reduces the total TAU [45]. FBXL20 is a protein–ubiquitin ligase, a member of the F-box protein family, known to be involved in neural transmission. The knockdown of *FBXL20* in the hippocampus of a depressed rat model led to an increase in the VGLUT1 and VAMP1 synaptic proteins, improving synaptic transmission and depression-like behavior [46]. Another gene involved in ubiquitin-mediated degradation is *FBXW4*, a member of the F-box/WD-40 gene family. Lastly, BCL2L13 is a BCL2-like protein localized in mitochondria that may promote a pro-apoptotic function, mitophagy, and mitochondrial fragmentation [47]. We did not find any direct correlation between AD and the other genes.

Regarding lncRNA analysis, we obtained a total of 20 different antisense lncRNA genes mapping to 18 DASE regions. Interestingly, again, *MAPK10* and *CHRNA7*—Alzheimer’s disease pathway-related genes—were recognized as the putative targets of specific brain-expressed lncRNAs (Table 5 and Table S6).

Notably, we specifically focused on shared DASEs to understand the potential molecular mechanisms of CBNR in resolving the specific aberrant splicing pattern resulting from Aβ exposure. However, other genes and thus other processes have also been impacted by alternative splicing in both the Aβ and CBNR pre-treatment conditions (Figure 2), which could collectively aid in the neuroprotection of neurons. Follow-up studies may help to clarify the contribution of the total splicing pattern generated by Aβ exposure and CBNR pre-treatment. Another issue that could be tackled in future studies is to investigate the molecular mechanisms underlying the generation of splicing aberrancy, for instance, studying the occurrences of RNA-binding proteins (including splicing factors) around the genomic region of DASEs. Furthermore, we emphasize that the analyses on DASE/miRNA hybridizations and DASE/lncRNA mapping are predictive analyses and thus require dedicated validation approaches aimed at verifying the expression of these ncRNAs and their regulation on putative target mRNAs. Nevertheless, our analysis provides guidance for further research, offering insights into how splicing regulation and downstream post-transcriptional processes are closely interconnected in ultimately regulating gene expression. Finally, in this study, we followed a standardized workflow (e.g., [48,49,50,51]) using widely adopted tools like rMATS for event-based alternative splicing analysis. Throughout the study, we consistently opted for stringent parameters to highlight the most significant events, processes, and predictions, thereby minimizing the background noise that is commonly present in this type of analysis.

In conclusion, we documented for the first time that a cannabinoid, CBNR, is able to regulate AS in an in vitro AD model. CBNR pre-treatment restored the splicing defects produced by Aβ exposure, involving genes also highly associated with AD. Moreover, thanks to this mechanism, CBNR probably counteracts the Aβ-induced mis-regulation of genes, due to premature stop codons and miRNA or lncRNA targeting. This work improves our knowledge of the molecular mechanisms that can be potentially useful in treating AD, corroborating the fact that drugs targeting post-transcriptional splicing processes could be considered novel and valid choices in neuroprotection and prevention issues.

## 4. Materials and Methods

### 4.1. Cell Culture, Treatment and Transcriptomic Analysis

In a previously published study by Chiricosta et al. [24], our research group cultured SH-SY5Y human neuroblastoma cell line and differentiated them with 10 µM retinoic acid (RA) for 5 days. Differentiated SH-SY5Y cells were pretreated for 24 h with 20 µM CBNR and then exposed to 10 µM β-amyloid peptide 1–42 (Aβ) for a further 24 h (CBNR20+Aβ sample). Another two conditions were investigated: differentiated SH-SY5Y cells exposed to 10 µM Aβ for 24 h (Aβ sample) and differentiated cells simply treated with PBS-diluted DMSO in the maintenance medium (CTR sample). The total RNA of the CBNR20+Aβ, Aβ, and CTR samples was extracted and subsequently sequenced in paired-end mode using the Illumina NextSeq™ 550Dx platform (Illumina, San Diego, CA, USA). Differentially expressed genes were identified with DESeq2 [52] by comparing the CBNR20+Aβ samples against the Aβ samples (CBNR20+Aβ vs. Aβ) and the Aβ samples against the CTR samples (Aβ vs. CTR), with q-value adjustment using the Benjamini–Hochberg method (threshold = 0.05).

### 4.2. Gene Ontology Analysis of DEGs

The resulting DEGs were used to perform a GO over-representation analysis (ORA) of biological processes (BPs) against the Homo sapiens reference. This well-established, statistical method was used to determine whether predefined sets of genes associated with specific biological processes are over-represented in our list of DEGs compared to what would be expected by chance [53]. The analysis was conducted using the PANTHER (V. 19.0) tool [54], available online at https://pantherdb.org (accessed on 1 January 2025), using the default parameters (Fisher’s exact test corrected by the false discovery rate). Splicing-related BPs were further analyzed with GOATOOLS (V. 1.4.12) to describe the formal relationships among these processes using attributes such as “is a” and “part of”, as well as to ultimately represent them in a directed acyclic graph (DAG).

### 4.3. Differential Alternative Splicing Events Analysis

Differential alternative splicing events were detected and quantified using the rMATS (replicate Multivariate Analysis of Transcript Splicing) computational tool [26], starting from read alignment (available at the NCBI Sequence Read Archive with accession number PRJNA1079210 [24]) to the well-annotated human reference genome GRCh38 deposited on Ensembl release 112 (accessed on 4 July 2024) [55]. The tool computed how often a specific exon was included in a particular region and expressed this measure as PSI (Percent Spliced In). Splicing events were indeed quantified using the PSI values ranging from 0 (which indicates that the exon is never included) to 1 (which indicates that the exon is always included). PSI count was based on the number of reads that unambiguously supported the inclusion of the exon and the number of reads that unambiguously supported the exclusion of the exon. After PSI computation, rMATS then compared the PSI values between the replicates and across experimental conditions to identify alternative splicing events where the PSI values were significantly different across the conditions (ΔPSI).

**Figure 8:**
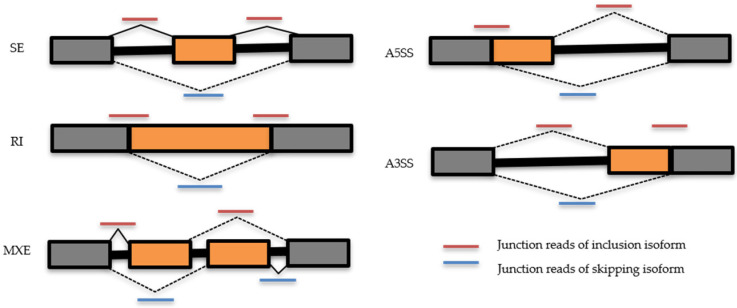
Scheme of ASEs (A3SS, A5SS, MXE, RI, SE) and usage of junction reads by rMATS tool (V. 4.3.0). In orange are represented the alternative cassette regions, in grey the flanked ones, for each ASE class. Lines in red represent the junction reads of inclusion isoform and in blue the ones of skipping isoform.

We retained only the significant DASEs with a false discovery rate (FDR) ≤ 0.05 and filtered events for 0.1 ≤ ΔPSI ≤ −0.1. Finally, since greater RNA-Seq read counts ensure a more reliable estimation of splicing events, a coverage threshold of 5 was applied for each event under investigation. Both the annotated and de novo splice sites were analyzed, and the resulting five types of splicing events are reported in Figure 8.

#### Shared DASEs Analysis

The outputs from both rMATS runs (one for CBNR20+Aβ vs. Aβ and the other for Aβ vs. CTR) were further analyzed with the final aim of retaining a set of common DASEs across the two. Therefore, we selected only a subset of genes that were differentially spliced in both comparisons (shared DASEs) and used as input to the PANTHER Pathways and PANTHER GO-Slim BP analyses (accessed on 1 January 2025). Both tools performed Fisher’s exact test with FDR correction using all Homo sapiens genes as a reference list, returning a list of pathways and biological processes characterized by an over-representation of shared DASEs.

The absolute PSI and ΔPSI values of the shared DASEs were described and plotted using a custom Python (V. 3.9.12) script that incorporated the pandas (V. 2.2.2) library for data manipulation, as well as matplotlib (V. 3.9.1) and seaborn (V. 0.13.2) packages for data visualization thus facilitating the exploration of the rMATS results while generating useful plots to visually represent and summarize our findings.

### 4.4. RI Premature Stop Codon Identification

The RI coordinates of the sequence were obtained from the original rMATS output, including the nucleotides between the upstream exon end and the downstream exon start. We manually inspected the RI fasta sequence for the appropriate ORF using the Integrative Genomics Viewer (IGV) [56], a high-performance, easy-to-use, interactive tool for the visual exploration of genomic data. Afterwards, we ran an in-house written python script that used the input RI fasta sequence and their associated reading frame to identify the premature stop codon (TAG, TAA, and TGA) if present. RI events identified in the *MED12* and *ENSG00000288694* genes were excluded from our premature stop codon analysis. This decision was based on the manual curation of the splicing events, where an inspection of the sequencing data through IGV revealed no evidence of retained introns at the coordinates reported by the rMATS output.

### 4.5. DASE/miRNA Hybridization Analysis

The miRNA–target binding configuration was predicted using the RNA-hybrid tool from the Bielefeld Bioinformatics Server [57], which determines the most favorable hybridization site between miRNAs and DASE sequences with a specific overall Minimum Free Energy (MFE). Both the miRNA and shared DASE fasta sequences were retrieved and used as input for the RNA-hybrid microRNA–target duplex prediction. Specifically, the miRNA sequences were downloaded from the microRNA gene database MirGeneDB 2.1 [58], which is publicly and freely available at http://www.mirgenedb.org/, comprising a list of 630 known miRNA sequences annotated in the human genome. For each type of splicing event, the nucleotide sequences were extracted according to specific guidelines outlined in the rMATS manual. For SE, we extracted the nucleotide sequences between the exon start and the exon end. In the case of MXE, the nucleotide sequence was determined by the strand orientation. For the positive strand, the sequence was taken between the first exon start and the first exon end. Conversely, for the negative strand, the sequence was between the second exon start and second exon end. For A3SS and A5SS, we extracted the nucleotide sequences between the long exon start and the long exon, ensuring that any overlapping sequence with the region between the short exon start and the short exon was excluded. Finally, for RI, we extracted the nucleotide sequences between the upstream exon end and the downstream exon start. Following these rules, the fasta sequences of the shared DASEs were therefore extracted directly from the human reference genome GRCh38 deposited on Ensembl, using the getfasta function of the bedtools utilities (V. 2.30.0) [29] and the DASE coordinates in a BED format. To properly run the RNA-hybrid miRNA–target prediction, an MFE threshold of −20 kcal/mol filter was applied for the identification of strong hybridizations [57].

#### miRNA-DASE Hybrids Classification and Filtering

The RNA-hybrid miRNA–target predicted alignments were ultimately classified based on seed and post-seed pairings in the “Canonical”, “Strong”, and “Compensatory” alignments [35,36]. The “Canonical” ones were characterized by a perfect match in the seed region (positions 2–7) with no additional pairings in the post-seed region. The “Strong” alignments were characterized by both seed pairing and additional complementary pairing in the post-seed region (positions 13–16). Finally, miRNA–target predicted alignments were classified as “Compensatory” if at least one mismatch in the seed position was present but complementary binding existed in the post-seed region. We only considered Watson–Crick pairing between the miRNA and the target for matching positions, so guanine (G) must pair with cytosine (C), and adenosine (A) with uracil (U). Therefore, whenever a G was found to pair with a U, it was considered a mismatch. Moreover, for each resulting hybridization, we annotated whether an A opposite of position 1 of the miRNA was present, since it is known to facilitate target recognition by the Argonaute proteins [35]. Based on this classification, we decided to exclude the predicted hybridizations that did not fall under any of the 3 classes. The resulting hybrids were filtered based on their MFE, which was required to be lower than −30 kcal/mol to ensure the selection of only strong interactions, as described by Alves Jr et al. [59].

The final filter applied to this last set of miRNA-DASEs interactions was based on the presence and expression levels of miRNAs in the brain. To achieve this, we accessed the publicly available database miRNATissueAtlas2 [60,61] (accessed on 13 November 2024), which comprises NGS data on sncRNAs and their expression across various tissues. To retain only the interactions involving miRNAs with elevated expression in the brain, as identified by the NGS data, we first screened the miRNATissueAtlas2 database by organ and tissue type. We then obtained a list of miRNAs expressed in the “brain”, along with their expression values. This tissue-specific subset of the miRNATissueAtlas2 database was used to identify a range of elevated expression values as those between the 90th percentile and the maximum observed expression. Finally, we subsetted our list of miRNAs hybridized with DASEs to include only the ones that had an associated expression value in the brain within this high-expression range. To ultimately check whether the resultant miRNAs were already associated with Alzheimer’s disease, we referred to the publicly available miRNA–disease associations database deposited by Madan et al. at https://zenodo.org/records/10523046 (accessed on 12 November 2024) [38]. All the retained DASE/miRNA hybridizations were annotated with an AD association score as reported in database, with the relative reference PMID (Table S5).

### 4.6. lncRNA Mapping to DASE

The lncRNA mapping on the identified DASEs was performed with reference to a comprehensive annotation file of lncRNA coordinates from the LncBook 2.0 database. This database integrates human lncRNAs with multi-omic annotations, comprising 95,243 different lncRNA genes and 323,950 different lncRNAs transcripts, thus providing a robust reference for our analysis [28]. The IntersectBed function of the bedtools utilities (V. 2.30.0) [29] was then used to check the overlaps between the genomic coordinates of the DASEs and the lncRNA annotations with the imposition of different strand constraints to selectively check for antisense sequences.

The NONCODEv4 database was iteratively browsed to check for lncRNA expression in brain tissue [62], as it collects lncRNA expression patterns across various tissues. The resulting antisense lncRNAs mapping to DASE regions were ultimately annotated based on whether they were reported in the database as being expressed in brain tissue or not (Table S6).

### 4.7. Sashimi Plot Visualization

Rmats2sashimiplot v3.0.0, provided by Zhijie Xie and freely available on github at https://github.com/Xinglab/rmats2sashimiplot (accessed on 27 May 2024), was used to produce sashimi plot visualizations of the rMATS outputs. For each comparison, it considered the input of the Binary Alignment Map (BAM) of each sample, which was the comprehensive compressed binary representation of the Sequence Alignment Map files, and the annotation file of the genomic coordinates of the human reference genome GRCh38 in the Generic Feature Format (gff3 file). Two different runs of Rmats2sashimiplot were executed, one for each comparison—CBNR20+Aβ vs. Aβ and Aβ vs. CTR. The first one involved the four BAM files, one for each of the two replicates of the CTR samples and the two replicates of the Aβ samples, together with the GRCh38 gff3 annotation file. The second one involved the four BAM files, one for each of the two replicates of the Aβ samples and the two replicates of the CBNR20+Aβ samples; together with the GRCh38 gff3 annotation file. For both runs, the input mapping files (BAM files) were divided into two different groups for plotting to include replicates of the same condition in the same group while calculating an average inclusion level, an average read depth, and an average number of junction-spanning reads for each group. Rmats2sashimiplot ultimately produced sashimi plots, a graphical representation to quantitatively visualize splice junctions for the mRNA sequences aligned to an annotated genomic reference, which allowed us to screen the shared DASEs identified.

## Figures and Tables

**Table 1:** List of the most enriched biological processes for both Aβ vs. CTR and CBNR20+Aβ vs. Aβ.

GO Biological Process (BP)	CBNR20+Aβ vs. Aβ Fold Enrichment	CBNR20+Aβ vs. Aβ FDR	Aβ vs. CTR Fold Enrichment	Aβ vs. CTR FDR
regulation of DNA-templated transcription elongation	3.1	0.00108	2.25	0.0118
RNA splicing, via transesterification reactions	2.08	0.00985	1.85	0.00272
mRNA splicing, via spliceosome	2.05	0.0147	1.88	0.00236
RNA splicing, via transesterification reactions with bulged adenosine as nucleophile	2.05	0.0145	1.88	0.00235
mRNA processing	1.98	0.000156	1.78	0.0000267
RNA splicing	1.92	0.00386	1.82	0.0000962
RNA processing	1.75	0.0000119	1.52	0.0000751

Table displays the top 10% of enriched BPs, ordered for fold enrichment. Six out of seven BPs are related to splicing mechanism.

**Table 2:** The only enriched biological process in Aβ vs. CTR and CBNR20+Aβ vs. Aβ.

PANTHER Pathways	Genes	Fold Enrichment	Raw *p* Value	FDR
Alzheimer disease-amyloid secretase pathway	*ADAM9*, *APBA2*, *CHRNA7*, *MAPK10*, *PRKCI*	19.25	6.17 × 10^−6^	9.87 × 10^−4^

“Alzheimer’s disease–amyloid secretase pathway” is characterized by a significant over-representation of genes involved in shared DASEs.

**Table 3:** The table reports RI coordinates, with the correct ORF identified, the number of premature stop codons encountered, and the position of the first stop codon identified.

Gene	RI Coordinates	Frame of ORF	n. of Premature Stop Codons	Position of the First Stop Codon
*ADAM9*	chr8:39018918-39021642 (+)	Frame 1	59	73
*DDX39A*	chr19:14409447-14409535 (−)	Frame 2	2	2
*GGA3*	chr17:75240108-75240341 (−)	Frame 1	3	19
*HAX1*	chr1:154273598-154273773 (+)	Frame 3	2	57
*LIG3*	chr17:34991837-34991957 (+)	Frame 2	1	41
*MAPK10*	chr4:86098595-86101051 (−)	Frame 3	57	39
*MROH1*	chr8:144260544-144260676 (+)	Frame 1	No stop codons	---
*NECAP1*	chr12:8091850-8092675 (+)	Frame 2	12	2
*PDHB*	chr3:58431793-58431876 (−)	Frame 1	No stop codons	---
*POLG*	chr15:89318749-89318930 (−)	Frame 1	1	130
*TBC1D20*	chr20:441689-441856 (−)	Frame 2	3	8
*ENSG00000271793*	chr6:85547397-85547505 (−)	Frame 1	1	40
*ENSG00000284946*	chr15:90981033-90981498 (−)	Frame 1	16	25

**Table 4:** List of miRNA–target hybridizations predicted by RNAhybrid tool, having a MFE ≤ −30 and involving miRNA sequences highly expressed in brain tissue.

ASE Class	Gene	miRNA	ΔPSI Aβ vs. CTR	ΔPSI CBNR20+Aβ vs. Aβ	MFE (kcal/mol)
A5SS	*POLR2J3*	hsa-miR-134-5p	0.136	−0.127	−30.3
RI	*ENSG00000284946*	hsa-miR-877-5p	0.113	−0.12	−31.9
RI	*NECAP1*	hsa-miR-628-3p	0.194	−0.159	−32.5
RI	*MAPK10*	hsa-miR-744-5p	−0.217	0.244	−40.5
RI	*POLG*	hsa-miR-328-3p	0.278	−0.233	−37.1
RI	*POLG*	hsa-miR-423-3p	0.278	−0.233	−38.4
RI	*POLG*	hsa-miR-874-3p	0.278	−0.233	−35.7
RI	*POLG*	hsa-miR-1249-3p	0.278	−0.233	−32.8
RI	*MROH1*	hsa-miR-328-3p	−0.351	0.351	−34.8
RI	*MROH1*	hsa-miR-331-3p	−0.351	0.351	−34.8
RI	*MROH1*	hsa-miR-874-3p	−0.351	0.351	−35.5
A3SS	*TYK2*	hsa-miR-328-3p	−0.458	0.458	−34.1
A3SS	*TYK2*	hsa-miR-874-3p	−0.458	0.458	−42.4
A3SS	*TYK2*	hsa-miR-1249-3p	−0.458	0.458	−32.9
A3SS	*TYK2*	hsa-miR-3200-3p	−0.458	0.458	−31.3
A3SS	*GTF2IRD1*	hsa-miR-185-5p	−0.328	0.221	−30.7
SE	*FBXL20*	hsa-miR-671-5p	−0.15	0.171	−32.6
SE	*APBA2*	hsa-miR-145-5p	−0.134	0.151	−30
SE	*APBA2*	hsa-miR-328-3p	−0.134	0.151	−31.7
SE	*APBA2*	hsa-miR-370-3p	−0.134	0.151	−41
SE	*APBA2*	hsa-miR-744-5p	−0.134	0.151	−39.6
SE	*APBA2*	hsa-miR-1301-3p	−0.134	0.151	−30.9
SE	*CHRNA7*	hsa-miR-149-5p	−0.242	0.242	−33.3
SE	*FBXW4*	hsa-miR-874-3p	−0.13	0.146	−35.4
SE	*HIP1*	hsa-miR-145-5p	−0.149	0.149	−32
SE	*HIP1*	hsa-miR-423-3p	−0.149	0.149	−35.3
SE	*HIP1*	hsa-miR-1180-3p	−0.149	0.149	−32.5
SE	*LAMB1*	hsa-miR-744-5p	−0.167	0.151	−31.1
SE	*BCL2L13*	hsa-miR-185-5p	−0.209	0.246	−31.4
SE	*ZC3H4*	hsa-miR-149-5p	−0.234	0.234	−31
SE	*ZC3H4*	hsa-miR-328-3p	−0.234	0.234	−31.1
SE	*MVK*	hsa-miR-652-3p	−0.297	0.297	−30.2
SE	*MVK*	hsa-miR-874-3p	−0.297	0.297	−34.3

**Table 5:** List of lncRNAs mapped to shared DASEs and expressed in brain tissue.

ASE Class	DASE Gene Name	ΔPSI Aβ vs. CTR	ΔPSI CBNR20+Aβ vs. Aβ	lncRNA Gene_ID
MXE	*MAPK10*	−0.229	0.229	*HSALNG0035653*
RI	*ENSG00000284946*	0.113	−0.12	*HSALNG0108169*
RI	*PDHB*	0.114	−0.131	*HSALNG0026424*
SE	*ZNF468*	0.168	−0.21	*HSALNG0127347*
SE	*CHRNA7*	−0.242	0.242	*HSALNG0104832*

## Data Availability

The data presented in this study are openly available in the NCBI Sequence Read Archive at BioProject, accession number PRJNA1079210.

## Supplementary Materials

The following supporting information can be downloaded at: https://www.mdpi.com/article/10.3390/ijms26073113/s1.

## Abbreviations

The following abbreviations are used in this manuscript:

AD	Alzheimer’s disease
A3SS	alternative 3′ splice site
A5SS	alternative 5′ splice site
AS	alternative splicing
Aβ	β-amyloid
BP	biological process
CBD	cannabidiol
CBG	cannabigerol
CBNR	cannabinerol
DAG	directed acyclic graph
DASE	differential alternative splicing event
DEG	differentially expressed gene
FDR	false discovery rate
GO	Gene Ontology
GWAS	genome-wide association studies
IGV	Integrative Genomics Viewer
lncRNA	long non-coding RNA
MFE	Minimum Free Energy
miRNA	microRNA
MXE	mutually exclusive exon
ncRNA	non-coding RNA
NMD	nonsense-mediated decay
ORA	over-representation analysis
ORF	open reading frame
PSI	Percent Spliced In
RA	retinoic acid
RI	retained intron
rMATS	replicate Multivariate Analysis of Transcript Splicing
SE	skipped exon
sncRNAs	small non-coding RNAs
Δ^8^-THC	∆^8^-tetrahydrocannabinol
Δ^9^-THC	Δ^9^-tetrahydrocannabinol
