# Supplementary figures and images for: Cannabinerol Restores mRNA Splicing Defects Induced by β-Amyloid in an In Vitro Model of Alzheimer’s Disease: A Transcriptomic Study
Source: Int J Mol Sci. 2025 Mar 28;26(7):3113. doi: 10.3390/ijms26073113 (PMC11988423; doi:10.3390/ijms26073113)

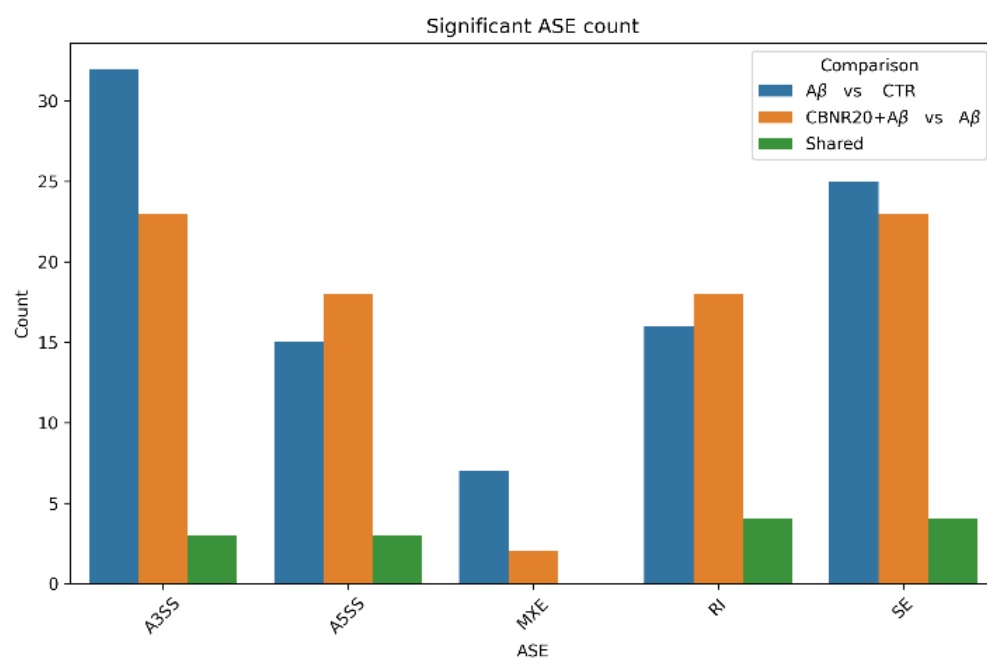

**Figure S1.** De-novo DASE count for each category resulting from rMATS analysis

Supplement: Supplementary file 1 [file ijms-26-03113-s001.zip › Figure S1.pdf]
